# Characteristics of the Pressing Process and Density Profile of MUPF-Bonded Particleboards Produced from Waste Plywood

**DOI:** 10.3390/ma17040850

**Published:** 2024-02-10

**Authors:** Agnieszka Laskowska

**Affiliations:** Institute of Wood Sciences and Furniture, Warsaw University of Life Sciences, 159 Nowoursynowska St., 02-776 Warsaw, Poland; agnieszka_laskowska@sggw.edu.pl

**Keywords:** density profile, particleboard, pressing, recovered particles, recycling, resin

## Abstract

Waste plywood containing phenol–formaldehyde (PF) resin is one of the materials that are difficult to use in the production of particleboards based on UF resin. Therefore, the aim of this research was to analyze the possibility of using this type of waste in the production of particleboards bonded with melamine-urea-phenol-formaldehyde (MUPF) resin in order to determine their suitability for particleboard production. The pressing process and density profile of three-layer particleboards were presented. The press closing time for mats containing only recovered particles in the core layer (100%), produced with a face layer ratio of 50%, a resin load for a face layer of 12%, and a core layer of 10%, at a unit pressure of 3 MPa, was 29% shorter than for the industrial particle mats. Regardless of the level of variability of independent factors, the heating time of the mats containing recovered particles was 10–20% shorter than the heating time of the mats with industrial particles. The greatest impact on the maximum density of the face layer of particleboards was observed for the content of the recovered particles and then the resin load. The maximum density area of the face layer was located closer to the surface in particleboards produced with a higher (80%, 100%) content of the recovered particles, a higher (i.e., 12% and 10%, respectively, for face and core layers) resin load, a lower (35%) face layer ratio, and a higher (3 MPa) unit pressure.

## 1. Introduction

The generation of various types of residues is an inseparable phenomenon that accompanies the woodworking process. According to the waste hierarchy indicated in the Directive 2008/98/EC of the European Parliament and of the Council of 19 November 2008 on waste and repealing certain Directives [1] in the field of waste management, firstly, it is necessary to prevent the generation of waste, then prepare for reuse and recycling and only at the end implement other recovery methods, i.e., energy recovery or disposal. It should be noted that energy recovery and the reprocessing into materials that are to be used as fuels or for backfilling operations, such as collecting waste in landfills, are not a forms of recycling.

There are a number of factors that distinguish a given type of board from a group of wood materials. An important parameter in relation to particleboards is the resin load, or, in relation to plywood or LVL, the glue rate. In the case of particleboards, the resin load is 8–10%, while in the case of medium density fibreboard (MDF), it is 8–12% [2]. Amino resins, i.e., urea–formaldehyde (UF) resin and melamine–urea–formaldehyde (MUF) resin, are usually used for the production of particleboard and MDF. From groups of layered materials, plywood is produced and used in most European countries, while laminated veneer lumber (LVL) is used in Scandinavian countries, Canada, and the United States. Depending on the purpose, plywood is made on the basis of UF or PF resin, and LVL mainly uses PF and melamine–formaldehyde (MF) resins. The PF resins are the first synthetically obtained resins to be used on a large scale in technology. The process of obtaining PF resin is a classic polycondensation reaction (the reactants are phenol and formaldehyde), involving the splitting of a water molecule. The PF resins are thermosetting resins used in the production of wood-based panels for exterior applications [3]. The glue rate used in the production of plywood is in the range of 160–200 g·m^−2^ and in the production of LVL, 220–250 g·m^−2^ [4,5]. With the indicated application of the glue 160–200 g·m^−2^, its consumption is 110–140 kg·m^−3^, of which 70–90 kg·m^−3^ is resin, and the rest are other chemical agents (i.e., filler and hardener). Calculated on the weight of completely dry wood, the dry weight of the resin is 12–15% [6]. The fact that layered materials contain the most resin, e.g., UF, is reflected in the nitrogen content in the interior plywood waste [7].

Amino and phenolic synthetic resins are not classified as hazardous agents. Only the formaldehyde formed during the decomposition, mainly of UF resins, is classified as a category 3 carcinogen, i.e., a substance with a possible carcinogenic effect on humans, as there is limited evidence of such an impact [8]. On this basis, the permissible content of formaldehyde in the boards or its emission from the boards was determined. According to EN 13986:2004+A1:2015 [9], wood materials should be produced in the formaldehyde emission class E1 or higher, Super E0. Wood-based panels are also produced in the E0.5 emission class. Therefore, wood waste, both industrial as well as post-consumer products containing synthetic resins, does not belong to hazardous materials and can be subjected to various forms of recovery.

Knowledge about the possibility of managing waste wood, in particular “contaminated” with synthetic resins, in particleboard production is incomplete [10]. Czarnecki et al. [11] tested in laboratory conditions the possibility of replacing industrial particles of the core layer of three-layer particleboards with wood particles from wood waste (UF- and PF-bonded raw and laminated particleboards and UF-bonded MDF). Demirkir and Çolakoğlu [12] examined formaldehyde emissions from particleboards manufactured with waste veneers, plywood edgings, and veneer peeler cores. Kurowska et al. [13] conducted laboratory tests on the possibility of using particles from waste plywood contaminated with UF resin to produce OSB. Andrade et al. [14] examined the strength properties and dimensional stability of particleboards with different proportions of thermally treated recycled pine particles. Lee et al. [15] stated that wood particles are the second-most costly element after resin in particleboard production, where both elements account for more than 50% of the overall production cost. Therefore, it is justified to take steps to replace industrial particles with recovered particles.

There is a lack of data on the pressing process of mats containing recovered particles that are “contaminated” with resin and the characteristics of the density profile of the particleboards. The pressing operation is the most important stage of the particleboard production process. It determines the physical and mechanical properties of the particleboards [16,17]. During the pressing process, five phases of temperature changes inside the mat can be distinguished [18,19]: I—heat transfer from the press heating plates to the core of the mat (no temperature increase); II—heating the core layer of the mat until the water begins to evaporate (rapid temperature increase); III—evaporation of water until the boiling point of water inside the mat is reached (slow temperature increase); IV—steam escape from the mat (constant temperature); and V—further heating caused by heat conduction through the particles (very slow temperature increase). In the process of pressing particleboards, the heating of the mat usually ends in phase IV. Taking into account changes in the pressing pressure course, the following phases can be distinguished [6,20]: I—closing the press; II—pressure increase (pressing the mat to the required thickness); III—maintaining the maximum pressure; IV—pressure reduction; V—unloading the mat (pressure reduction to zero); and VI—opening the press.

It is generally assumed that the course of the mat pressing operation has a significant impact on the density distribution on the cross-section of the particleboards and, consequently, on their properties [21,22,23]. In the literature, the most frequently analyzed parameters of the density profile are the maximum density of the face layer, the minimum density of the core layer, and the distance between the maximum density area of the face layer and the particleboard surface [22,24]. In industrial conditions, an important indicator defining the “correct” density profile is the ratio of the minimum density of the core layer to the medium density of the particleboard. It is generally assumed that it should not be less than 85%. The relationships between the course of the pressing operation and the density profile of the particleboards constitute the basis for the analysis of phenomena shaping the physical and mechanical properties of particleboards [25,26]. The problem of using recycled particles in the production of particleboards is rarely analyzed. The research conducted by Laskowska and Mamiński [6] shows that PF-covered particles are poorly bondable materials in comparison to the UF-covered ones in UF-bonded particleboards. Therefore, there is a need to verify the possibility of using this type of waste in the production of MUPF-bonded particleboards in order to determine their suitability for particleboard production. There is a lack of data on the characteristics of the pressing process and mats containing recycled particles. For this reason, the aim of this research was to analyze the pressing process and selected properties of mats containing recovered particles.

## 2. Materials and Methods

### 2.1. Waste Wood with Synthetic Resin

The subject of this research was post-industrial waste plywood containing synthetic resin. The waste PF-bonded pine (*Pinus sylvestris* L.) plywood was obtained from two plants in Poland. The material in the amount of 0.5 tons was generated in edging operations. The plywood specifications were as follows: thicknesses of 7 mm, 12 mm, 16 mm, and 22 mm; a density of 660 kg·m^−3^; and a moisture content of 6%. The plywood was composed of an odd number of veneers that were 1.4 mm, 1.8 mm, 2.2 mm, or 2.5 mm thick. The industrial binder formulations contained about 30% solid additives, i.e., rye flour. A total of 1 m^3^ of plywood contained 75 kg of binder, which made up ca. 14% of the dry wood weight.

### 2.2. Particles from Waste Plywood

The shredding of waste plywood was carried out in accordance with the methodology presented by Laskowska and Mamiński [10]. A wood shredder with 10 mm, 14 mm, 25 mm, and 38 mm mesh screens and a constant knife–counter knife gap of 2.21 mm was used. The particles shredded on a 14 mm mesh screen in a single-shaft shredder exhibited properties (fractional composition, dimensions, and particle thickness distribution) closest to those found for industrial virgin particles in the core layer. Therefore, this type of recovered particle was used in the study. The face and core layers of the industrial particles were obtained from a particleboard plant in Poland. Their fractional compositions were typical for industrial applications [10].

### 2.3. Particleboard Preparation

Three-layer particleboards with dimensions of 16 mm × 320 mm × 320 mm (thickness × width × length) and a density of 650 kg·m^−3^ were prepared. The particleboards were bonded with MUPF resin. The MUPF resin used for the tests, in a normal climate (temperature of 20 ± 2 °C; relative humidity of 65 ± 5%), was characterized by a viscosity of 390 mPa s (100 rpm; spindle 64). Viscosity was determined using a Brookfield digital viscometer. The dry matter content of the MUPF resin was 65%, according to EN 827:2005 [27]. The adhesive formulation is presented in Table 1.

The choice of a particular variant of the adhesive formulation was made on the basis of its gel time, which was set at 210–220 s for the face layers and 110–120 s for the core layer of the mat. The gel times of the resins for the mat were selected to enable the curing of the glue in the center of the mat within the specified pressing time and, at the same time, prevent the glue from curing in the face layers before reaching the assumed board thickness. These conditions were met by the adhesive formulation that is given in Table 1.

The production of particleboards was carried out based on the Taguchi plan. The experimental plan for the production of particleboards is presented in Table 2. Conducting an experiment based on the assumptions of the adopted method enabled us to obtain repeatable and reliable test results. Moreover, it is possible to predict, determine, and define the interactions between factors [28]. A regular analysis of variables, based on the Taguchi method, includes a number of stages. As part of this work, the impact of selected factors, the so-called independent factors from the technological and material groups, on the particleboard properties and interactions between the factors were characterized.

The face layer ratio was set at 35% and 50%, which means that the face/core/face layer ratio was set at 17.5%/65%/17.5% and 25%/50%/25%. MUPF-resin load was 12% and 10%, respectively, for the face and core layers, or, in another variant, 10% and 8%, respectively, for the face and core layers. The contents of the recovered particles in the core layers were 20%, 60%, or 100%. Only the boards made of industrial particles were used as a reference. Then, sixteen variants of the boards were produced. Nine particleboards of each variant were prepared. The parameters of the hot pressing mats were as follows: unit pressure was set at 2 MPa or 3 MPa (Table 2), platen temperature was 180 °C, and time was 300 s.

### 2.4. Particleboard Properties

#### 2.4.1. Pressing Process Parameters

The mat pressing process was performed using a computer-controlled press. The mat core temperature, pressure, and thickness were monitored in real time throughout the pressing process for each mat using the computer controller. The mat core temperature, pressure, and thickness were determined with an accuracy of ±0.01 °C, ±0.01 MPa, and ±0.01 mm, respectively. The temperature in the mat core was monitored using an Fe-CuNi thermocouple.

#### 2.4.2. Density Profile

The particleboard density (MD) was determined according to EN 323:1993 [29]. The analysis of the particleboard density profile was based on the following four parameters: the maximum density of the face layer (DMax_FL_), the distance between the maximum density area of the face layer and the particleboard surface (ADMax_FL_), the minimum density of the core layer (DMin_CL_), and the ratio of the minimum density of the core layer to the medium density (DMin_CL_/MD).

The distribution of density over the thickness of the particleboard samples was determined using a laboratory device for measuring density profiles (Laboratory Density Analyzer DA-X) manufactured by GreCon Inc. (Tigard, OR, USA), which determines the density using X-rays. The tests were conducted on samples with a length and width of 50 mm × 50 mm at a measurement speed of 0.05 mm·s^−1^. Density values were measured every 0.02 mm of the thickness of the particleboard samples.

### 2.5. Statistical Analysis 

The statistical analysis of results was carried out with the use of the STATISTICA Version-13.3 software of StatSoft, Inc. (TIBCO Software Inc., Palo Alto, CA, USA). The analysis was based on the *t*-test, or ANOVA (Fischer’s F-test), with a significance level *p*-value of 0.050. On the basis of the sum of squares (SS), the percentual impact (the so-called factor influence) of the analyzed factors (i.e., content of the recovered particles, resin load, face layer ratio, and unit pressure) on the particleboard properties was calculated. The boards made of only industrial particles were used as a reference (control). The statistical analysis of the differences between the experimental grades and the reference was carried out at a significance level of 0.050.

## 3. Results

### 3.1. Influence of Selected Factors on Pressing of Mats Containing PF-Covered Particles

**Figure 1:**
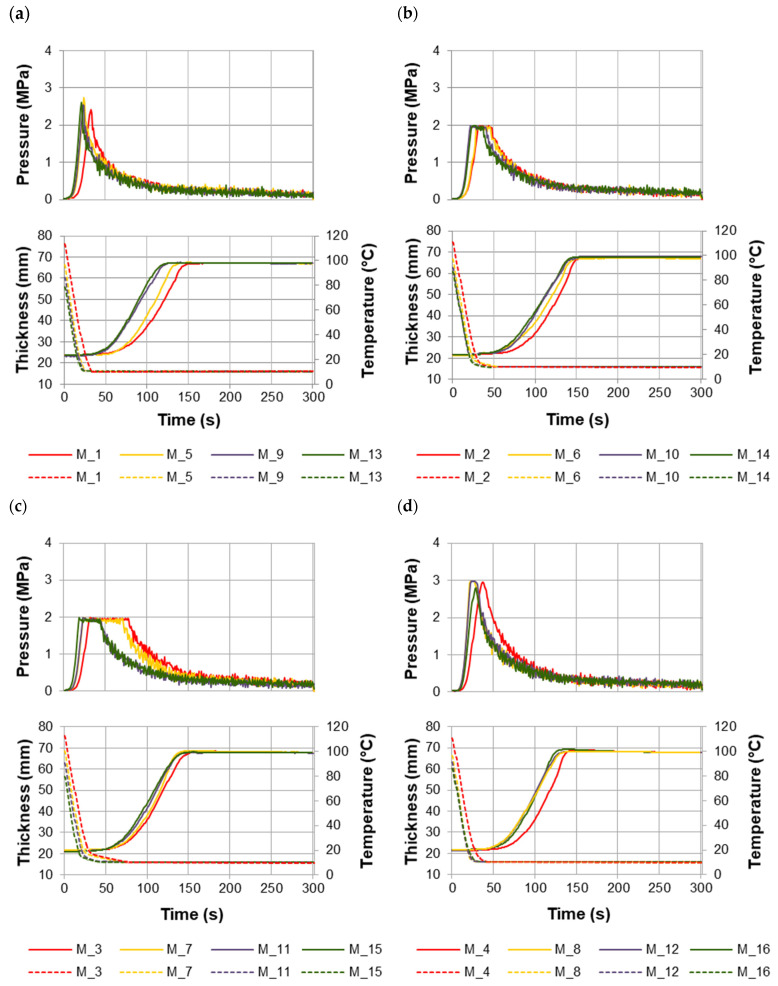
Pressure, mat core temperature, and mat thickness curves recorded in real time upon pressing: (**a**). M_1_5_9_13; (**b**). M_2_6_10_14; (**c**). M_3_7_11_15; (**d**). M_4_8_12_16.

Changes in the values of pressing pressure, temperature inside the mat, and thickness of the mat were shown in Figure 1. Due to the large group of tests (variants), it was divided into four subgroups that differ in the levels of independent factors. The markings in the legend and in the description of individual graphs refer to the sample number from the experimental design (Section 2.3, Table 2). The letter M was placed before the sample numbers as a symbol of the resin used for particleboard production.

Significant differences were found in the course of mat pressing, depending on the level of the independent factors. The greatest differentiation in the course of the pressing curves was observed in phase III—maintaining the maximum pressure—and in phase IV—pressure reduction (Figure 1). As a result, different press closing times were obtained. Differences in the curves of pressure, the mat core temperature, and mat thickness were visible during the pressing of the mats bearing recovered particles and the industrial particle mats (red line in the figure of each analyzed subgroup).

Out of the four subgroups of mats with recovered particles, the shortest press closing times were recorded during the pressing of mats at a unit pressure of 3 MPa, in which the face layer ratio was 50% and the MUPF-resin loads for the face and core layers were 12% and 10%, respectively (Figure 1a). In this subgroup, significant differences in the press closing times were noted between the industrial particle mats (M_1) and the mats with recovered particles in the entire ranges of 20%, 60%, and 100% (M_5, M_9, and M_13, respectively). However, there were no such differences in the press closing times between the mats containing 20% (M_5) or 60% (M_9) of recovered particles and the mats containing 100% of recovered particles in the core layer (M_13). For example, for the industrial particle mats (M_1), the press closing time was 35 s, and for mats containing only recovered particles in the core layer (M_13), it was 25 s, i.e., it was shorter by 29%.

The longest press closing times were obtained during the mats pressing at a unit pressure of 2 MPa, in which the face layer ratio was 50% and the resin loads for the face and core layers were 10% and 8%, respectively (Figure 1c). Under these conditions, the press closing time for the industrial particle mats (M_3) was 80 s, and for the mats with recovered particles in the entire ranges of 20%, 60%, and 100% (M_7, M_11, and M_15), the press closing times were 72 s, 45 s, and 45 s, respectively. This shows that an increase in the share of recovered particles resulted in a shortening of the press closing time by up to 40%.

The moisture contents of particles with a resin load of 12% for the face and 10% for the core layers were 14.5% and 12.5%, respectively. Whereas the moisture contents of particles with a resin load of 10% for the face and 8% for the core layer were 12.5% and 10%, respectively. Greater amounts of moisture in the mats made the wood more susceptible to elastic–plastic deformations, and as a result, it offered less resistance to the pressure exerted by the press shelves. During the pressing operation, greater amounts of water vapor were generated in the near-surface zones of the mat with higher moisture contents. As a result, water vapor moved more quickly to the center of the mat and then condensed there and released heat. This resulted in faster heating of the core layer of the mat. At the same time, the moisture of this layer increased, which resulted in the provision of “additional” amounts of heat needed to evaporate the moisture contained in it. The addition of recovered particles allowed for the faster transfer of water vapor and, thus, heat into the mat. This was related to the structure of the core layer of the mat containing recovered particles [30,31].

Simultaneously with the changes in the pressing pressure, changes in the mat core temperature occurred. Taking into account the phenomenology reported by Graser [18] and Bolton et al. [19], the greatest differences between the temperature curves in the core of individual mats occurred in phase II—heating of the layer in the mat core until the water began to evaporate. The shortest mat heating times were obtained when pressing mats at a unit pressure of 3 MPa, in which the face layer ratio was 50% and the resin loads for the face and core layers were 12% and 10%, respectively (Figure 1a). For this subgroup of mats, the shortest press closing times were also obtained. The heating time of the industrial particle mats (M_1) to a temperature of 100 °C was 150 s, and the mats with 20% (M_5), 60% (M_9), and 100% (M_13) of recovered particles were shorter by 10%, 20%, and 20%, respectively. In the remaining subgroups (Figure 1b–d), the heating time of the industrial particle mats to a temperature of 100 °C, similarly to the subgroup shown in Figure 1a, was approximately 150 s. However, the heating time of the mats in these subgroups, regardless of the content of the recovered particles (20–100%), was approximately 10% shorter than the time of heating the industrial particle mats.

The conducted research shows that the course of the mat pressing depends mainly on the level of the unit pressure and the content of the recovered particles. As a result of the greater pressure exerted on the pressed mat, it thickened faster. In the mats pressed at a higher (3 MPa) unit pressure, the water vapor generated moved to the core faster than in the mats pressed at a lower (2 MPa) pressure. The increase in the content of the recovered particles in the core layer of mats favored these phenomena. Due to the different dimensions and higher bulk density of recovered particles compared to industrial particles [10], the core layer of these mats was characterized by a larger amount of free space. Therefore, water vapor as an energy carrier had “easier” access to the center of the mat [19,32,33,34,35]. This resulted in shorter heating times for the mats and shorter times for achieving the assumed particleboard thickness. Additionally, the dynamics of mat heating could have been influenced by a higher resin load (i.e., 12% and 10%, respectively, for the face and core layers compared to lower resin loadsk i.e., 10% and 8%, respectively, for the face and core layers) resin load. Then, the particles in the mat had a higher moisture content, which resulted in a larger amount of water vapor generated during the pressing operation and a faster heat transfer to the center of the mat [6,33,36]. In general, it can be stated that regardless of the level of variability of the independent factors, i.e., resin load, face layer ratio, and unit pressure, the heating time of the mats containing recovered particles was 10–20% shorter than the heating time of the mats with industrial particles. It can also be generally assumed that regardless of the level of variability of factors, i.e., content of the recovered particles, resin load, and face layer ratio, the assumed thickness of particleboards manufactured at a pressure of 3 MPa was achieved in a time twice as short as in the case of particleboards manufactured at a pressure of 2 MPa.

### 3.2. Influence of Selected Factors on the Density Profile of Particleboards

The medium density (MD) of particleboards (all variants) was 655 kg·m^−3^. The obtained values were close to the board density of 650 kg·m^−3^ assumed in the tests. The medium density of individual particleboard variants (M_1–M_16) was in the range of 652–659 kg·m^- 3^. The differences between the densities of particleboards produced in different variants did not exceed 2%. Therefore, changes in the density of the particleboards should not affect their properties, and boards of different variants can be compared with each other. The analysis of the density profile of particleboards made with MUPF resin is presented in Table 3. The conducted research shows that the density of individual layers of particleboards varied significantly depending on the level of variability of the factors examined.

The maximum density of the face layer (DMax_FL_) of particleboards was in the range of 860–926 kg·m^−3^ (average 902 kg·m^−3^), with the lowest value recorded in M_15 and the highest in M_2 (Table 3). The area of maximum density of the face layers (ADMax_FL_) was located on average at a distance of 0.87 mm from their surface. The smallest distance (0.59 mm) was obtained by particleboards of variants M_9 and M_13, and the largest (1.28 mm) by M_3. The minimum density of the core layer (DMin_CL_) was in the range of 519–564 kg·m^−3^ (average 538 kg·m^−3^). The lowest DMin_CL_ value was for M_3, and the highest was for M_16. DMin_CL_ accounted for 83% of the MD of particleboards. The data in Table 3 show that only particleboards made in M_14 and M_16 DMin_CL_/MD achieved higher values than the required 85%.

**Figure 2:**
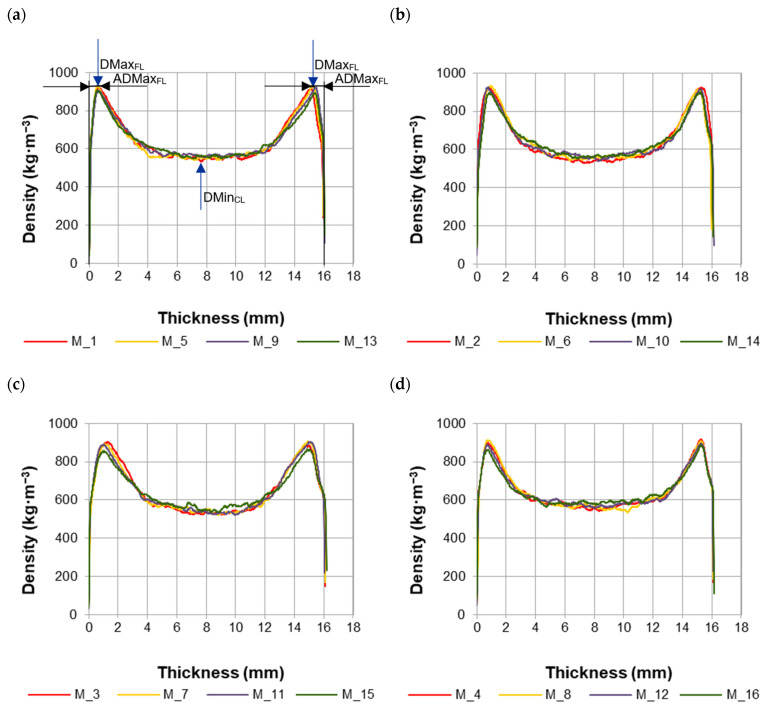
Density profiles of particleboards containing recovered particles: (**a**). M_1_5_9_13; (**b**). M_2_6_10_14; (**c**). M_3_7_11_15; (**d**). M_4_8_12_16.

Examples of density profiles of particleboards are shown in Figure 2. The group of particleboards was divided into four subgroups, differing in the levels of analyzed factors. The numbers in the legend of individual graphs refer to the sample number from the experimental plan (Section 2.3, Table 2). The letter M was placed before the sample numbers as a symbol of the resin used for particleboard production.

The density distribution on the cross-section of the particleboards had the shape of the letter “U” (Figure 2). The “borders” between the area of the face layers and the area of the core layer were clearly outlined. The greater the diversity of the density distribution on the cross-section of the particleboards, the higher the particles moisture and, thus, the shorter the heating time and compression of the mat to the assumed thickness. With more MUPF resin (higher resin loads, i.e., 12% and 10%, respectively, for the face and core layers compared to lower resin loads, i.e., 10% and 8%, respectively, for the face and core layers), greater amounts of water were introduced. The particles in the mat had a higher moisture content, which resulted in a larger amount of water vapor generated during the pressing operation and faster heat transfer to the center of the mat [33,36,37].

**Figure 3:**
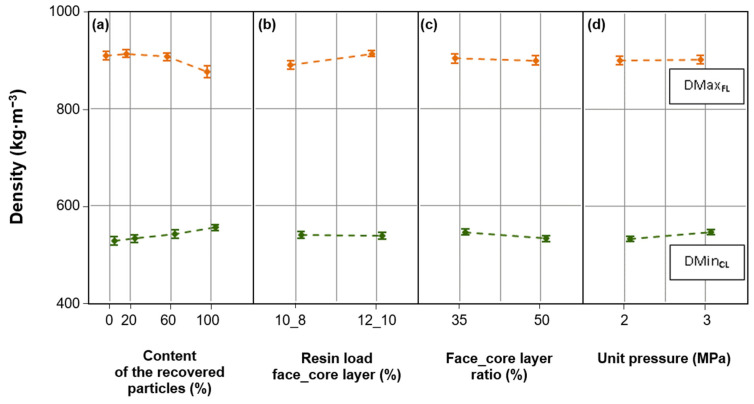
The maximum density of the face layer (DMax_FL_) and minimum density of the core layer (DMin_CL_) of particleboards depending on the: (**a**). content of the recovered particles; (**b**). resin load face_core layer; (**c**). face_core layer ratio; (**d**). unit pressure.

The research shows that the density of individual layers (face and core) of the boards was influenced by independent factors and their level. The results of the analysis of variance for selected factors and interactions between factors influencing DMax_FL_, ADMax_FL_, and DMin_CL_ of particleboards are presented in Table 4. The content of the recovered particles had the greatest impact (factor influence at the level of 52%) on the DMax_FL_ of particleboards. The resin load was the second-most important factor (factor influence at the level of 32%) influencing the DMax_FL_. It should be noted that the indicated factors were responsible for approximately 80% of the variability of DMax_FL_. The influence of the studied factors on the DMax_FL_ of particleboards is shown in Figure 3.

Figure 3a highlights that an increase in the content of the recovered particles resulted in lower DMax_FL_ values for the particleboards. The significant effect of the content of the recovered particles on the DMax_FL_ resulted mainly from the fact that recovered particles were characterized by a higher bulk density compared to industrial particles. These differences amounted to about 100 kg·m^−3^ (58%) and were statistically significant [10]. Depending on the content of the recovered particles in the core layer, the formed mats were 10–20 mm less thick compared to the mats of industrial particles (control variant). The temperature of 100 °C was reached faster inside the mats containing the recovered particles than in the mats containing industrial particles. This was due to the larger amount of free space between the recovered particles in the mats. Therefore, water vapor, as a carrier of thermal energy, moved deeper into the mat more quickly, and its core layer was heated faster [32,36]. As a result, the recovered particles “plasticized” faster than industrial particles. Therefore, these particles provided less resistance to the face layers of the particleboards than industrial particles, which resulted in lower DMax_FL_. The DMax_FL_ of particleboards made of industrial particles (M_1–M_4) was 910 kg·m^−3^, and of particleboards in which recovered particles accounted for 100% (M_13–M_16), it was 877 kg·m^−3^ (a decrease of 4%) (Figure 3a).

The resin load significantly influenced the DMax_FL_ of the particleboards (Figure 3b). The DMax_FL_ of particleboards containing larger amounts of MUPF resin (i.e., 12% and 10%, respectively, for face and core layers) was higher by 3% compared to the DMax_FL_ of particleboards containing smaller amounts of resin (i.e., 10% and 8%, respectively, for face and core layers). These relationships result from the fact that resins have a higher density than wood particles. Increasing the resin load in the particleboard was equivalent to reducing the amount of wood particles in the particleboard mass and, thus, in the particle mass of individual particleboard layers. As a result, higher DMax_FL_ values were obtained in particleboards made of particles with a higher resin load.

From the group of analyzed independent factors, the content of the recovered particles had the greatest impact on the DMin_CL_. The influence of this factor was 45%. The factors, the face layer ratio and the unit pressure, had a similar impact on the DMin_CL_ of the particleboards (19% and 15%, respectively). The data in Table 4 show that the conducted research failed to determine the causes of 18% of the DMin_CL_ variability in particleboards. It should be assumed that there are other factors, apart from those examined, that significantly influence DMin_CL_, e.g., press shelf temperature and pressing time. The influence of factors on the DMin_CL_ of particleboards is shown in Figure 3.

Figure 3a shows that an increase in the content of the recovered particles in the particleboards resulted in a higher DMin_CL_. Similarly to the previously described DMax_FL_ relationships, these changes were caused by the higher bulk density of the recovered particles compared to industrial particles and the course of the mat pressing. The recovered particles with a higher density, partially compacted, were subject to “additional” compression during the mat. The core layer of mats containing the recovered particles was heated faster, resulting in a greater degree of compaction. As a result, the DMin_CL_ of particleboards made with 100% of the recovered particles (M_13–M_16) was 5% higher than the DMin_CL_ of boards made from industrial particles.

The DMin_CL_ of the particleboards was significantly influenced by the face layer ratio (Figure 3c). The DMin_CL_ of particleboards made with a 35% share of face layers was 3% higher compared to the DMin_CL_ of particleboards made with a 50% share of face layers. A smaller (35%) share of face layers was equivalent to a larger (65%) share of the core layer of the particleboards. There were more recovered particles in this core layer of the particleboards. These relationships confirm the observations formulated in the context of analyzing the impact of the content of the recovered particles on the DMin_CL_.

It was shown that the particleboards were characterized by differences in DMin_CL_ depending on the level of unit pressure (Figure 3d). The DMin_CL_ of particleboards made at a pressure of 3 MPa was 3% higher than the DMin_CL_ of particleboards made at a pressure of 2 MPa. Depending on the level of the unit pressure, the press closing time varied. At a higher (3 MPa) mat pressing pressure, the press closing time was shorter than at a lower (2 MPa) pressing pressure. During the shorter press closing time, the particles of the face layers were rapidly compacted. However, the particles of the core layer resisted the denser particles in the face layers and, as a result, were less compressed. At a lower (2 MPa) mat pressing pressure, a longer press closing time was achieved, during which the mat was more uniformly heated. As a result, the particles of the core layer were more susceptible to compaction and offered less resistance to the particles in the face layers, which consequently resulted in a higher DMin_CL_.

**Figure 4:**
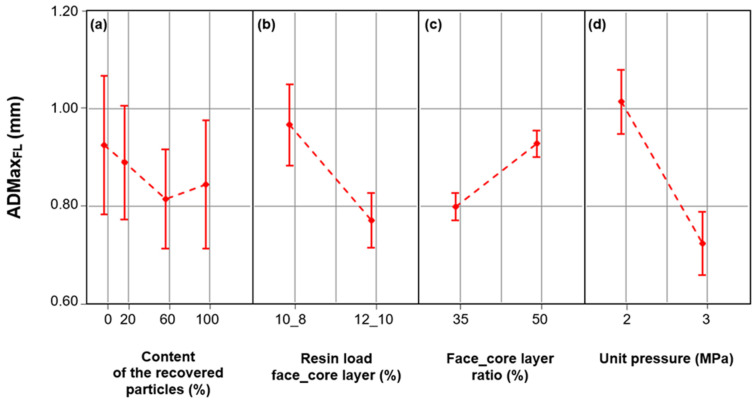
The distance between the maximum density area of the face layer and the particleboard surface (ADMax_FL_) depending on the: (**a**). content of the recovered particles; (**b**). resin load face_core layer; (**c**). face_core layer ratio; (**d**). unit pressure.

The conducted research shows (Figure 4) that all analyzed factors had a significant impact on the ADMax_FL_ of particleboards. The unit pressure had the greatest impact (58%) on the ADMax_FL_ (Table 4). The resin load (factor influence at the level of 26%) was the second-most important factor influencing the ADMax_FL_. The above-mentioned factors in total were responsible for approximately 80% of the variability of this value. In relation to ADMax_FL_, the following interactions were also statistically significant: content of the recovered particles x resin load and content of the recovered particles x face layer ratio (*p* < 0.050).

The increase in the amount of recovered particles in the core layer of the particleboards had a positive effect on ADMax_FL_ (Figure 4a). The literature data show that the greatest loads in a three-layer particleboard are carried by the face layers, and the closer to the surface of the particleboard, the greater its ability to bear loads [38]. The ADMax_FL_ of particleboards containing 20%, 60%, and 100% of recovered particles in the core layer was 4%, 13%, and 10% lower, respectively, compared to the ADMax_FL_ of particleboards made from industrial particles. As in the case of DMax_FL_, the presented dependencies resulted from the course of the mat pressing operation. The more recovered particles were in the core layer, the less thick the mats were. In mats with a smaller thickness, the densification of the face layers occurred faster than in mats of greater thickness, in which the particles of the core layer provided greater resistance to the particles of the face layers. As a result of the occurring phenomena, ADMax_FL_ was located closer to the surface in particleboards produced with a higher content of recovered particles.

Figure 4b shows that in particleboards containing larger amounts of MUPF resin (i.e., 12% and 10%, respectively, for the face and core layers), ADMax_FL_ was approximately 20% lower than in particleboards made with a smaller amount of resin (i.e., 10% and 8%, respectively, for the face and core layers). In particleboards with a higher resin load, a larger surface of adhesive joints was created and, thus, a more “compact” particle zone in the face layer. The zone of these particles offered greater resistance to the pressure exerted on the pressed mat than the zone of particles covered with a smaller amount of resin. This resulted in the formation of a larger DMax_FL_ moved closer to the particleboard surface.

The differences in the ADMax_FL_ values were also influenced by the face layer ratio (Figure 4c). In particleboards with a 50% share of face layers, ADMax_FL_ was 16% higher than in particleboards with a 35% share of face layers. The significant impact of the share of face layers on ADMax_FL_ resulted from the thickness they had in the mat. In mats with a larger (50%) share of face layers, there were fewer (50%) particles in the core layer compared to mats with a smaller (35%) share of face layers (share in the core layer of 65%). As a result, the particles of the core layer offered less resistance to the particles of the face layers, which resulted in a more uniform density of the particles of the face layers and the formation of a zone of maximum density of the face layers moving further from the particleboard surface.

The conducted research showed that the particleboards were characterized by differences in ADMax_FL_, depending on the level of the unit pressure (Figure 4d). Increasing the pressure during pressing the mats resulted in a lower ADMax_FL_. In particleboards manufactured at 3 MPa, ADMax_FL_ was 29% lower than in particleboards manufactured at 2 MPa. These dependencies were the result of the course of the pressing operation on the mats and the resulting press closing time [39,40]. As a result of the greater pressure (3 MPa) exerted on the pressed mat, the face layers rapidly thickened, resulting in lower ADMax_FL_. However, at lower pressing pressure (2 MPa), longer press closing times were achieved, which is equivalent to a longer time to obtain the assumed particleboard thickness. At lower pressure, during a longer press closing time, the entire mat gradually overheated, and thus the individual layers of particles became more evenly compacted. As a result, ADMax_FL_ in particleboards produced at a lower (2 MPa) pressing pressure was located further from the particleboard surface than in particleboards produced at a higher (3 MPa) pressing pressure. In the case of ADMax_FL_, interactions: content of the recovered particles × resin load for the face and core layers and the content of the recovered particles × face layer ratio were significant. However, these interactions showed differences only between ADMax_FL_ industrial particleboards. It should be assumed that the properties of recovered particles, i.e., a higher bulk density and a lower specific surface area than industrial particles, resulted in the “extinction” of the interactions in the case of ADMax_FL_-produced particleboards. In general, it can be concluded that ADMax_FL_ was located closer to the surface in particleboards produced with a higher (80% and 100%) content of the recovered particles, a higher (i.e., 12% and 10%, respectively, for the face and core layers) resin load, a lower (35%) face layer ratio, and a higher (3 MPa) unit pressure.

## 4. Conclusions

Post-industrial PF-bonded plywood is one of the most difficult-to-use materials in the production of particleboards produced with UF resin. Therefore, the aim of this research was to analyze the possibility of using this type of waste in the production of MUPF-bonded particleboards. Therefore, research was undertaken to verify the possibility of using this type of waste in the production of MUPF-bonded particleboards in order to determine their suitability for particleboard production. The pressing process and density profile of particleboards containing particles from post-industrial PF-bonded plywood were analyzed.

The shortest press closing times were recorded during the pressing of mats at a unit pressure of 3 MPa, in which the face layer ratio was 50% and the resin loads for the face and core layers were 12% and 10%, respectively. The press closing time for mats containing only recovered particles in the core layer was shorter by 29% than for industrial particle mats. Regardless of the level of variability of independent factors, i.e., the resin load, face layer ratio, and unit pressure, the heating time of mats containing recovered particles was 10–20% shorter than the heating time of the mats with industrial particles. The content of the recovered particles had the greatest impact on the maximum density of the face layer of particleboards and the minimum density of the core layer. All of the analyzed factors had a significant impact on the distance between the maximum density area of the face layer and the particleboard surface of particleboards. However, the unit pressure and the resin load in total were responsible for approximately 80% of the variability of this density parameter value. The analyses carried out will be implicated in further research. An important issue is determining the relationship between the pressing process, the density profile, and the physical and mechanical properties of particleboards with recovered particles.

## Figures and Tables

**Table 1:** MUPF adhesive formulation.

Component	Face Layer (ppw)	Core Layer (ppw)
MUPF	50.0	50.0
10% aq. (NH_4_)_2_SO_4_	3.0	7.0
water	12.0	8.0

**Table 2:** Experiment plan for the manufacture of particleboard with the addition of recovered particles.

Variant	Content of the Recovered Particles (%) in the Core Layer	Resin Load Face_Core Layer (%)	Face Layer Ratio (%)	Unit Pressure (MPa)
1	0	12_10	50	3
2	0	12_10	35	2
3	0	10_8	50	2
4	0	10_8	35	3
5	20	12_10	50	3
6	20	12_10	35	2
7	20	10_8	50	2
8	20	10_8	35	3
9	60	12_10	50	3
10	60	12_10	35	2
11	60	10_8	50	2
12	60	10_8	35	3
13	100	12_10	50	3
14	100	12_10	35	2
15	100	10_8	50	2
16	100	10_8	35	3

**Table 3:** Density profile parameters of particleboards with MUPF resin (DMax_FL_—the maximum density of the face layer; DMin_CL_—minimum density of the core layer; ADMax_FL_—the distance between the maximum density area of the face layer and the particleboard surface; DMin_CL_/MD—the ratio of the minimum density of the core layer to the medium density).

Variant	Content of the Recovered Particles (%)	Resin Load Face_Core Layer (%)	Face Layer Ratio (%)	Unit Pressure (MPa)	DMax_FL_	DMin_CL_	ADMax_FL_	DMin_CL_/MD
	(%)	(%)	(%)	(MPa)	(kg·m^−3^)	(kg·m^−3^)	(mm)	(-)
1	0	12_10	50	3	917 (7)	522 (10)	0.72 (0.04)	80 (1)
2	0	12_10	35	2	926 (3)	523 (11)	0.87 (0.07)	80 (2)
3	0	10_8	50	2	894 (4)	519 (6)	1.28 (0.06)	80 (1)
4	0	10_8	35	3	903 (5)	542 (12)	0.83 (0.03)	84 (2)
5	20	12_10	50	3	923 (12)	529 (12)	0.71 (0.03)	81 (2)
6	20	12_10	35	2	924 (10)	532 (10)	0.91 (0.09)	82 (1)
7	20	10_8	50	2	899 (3)	520 (3)	1.15 (0.06)	80 (1)
8	20	10_8	35	3	912 (6)	543 (9)	0.78 (0.06)	84 (1)
9	60	12_10	50	3	917 (4)	544 (12)	0.59 (0.05)	84 (1)
10	60	12_10	35	2	920 (4)	541 (5)	0.87 (0.02)	83 (1)
11	60	10_8	50	2	900 (10)	522 (3)	1.01 (0.02)	80 (2)
12	60	10_8	35	3	895 (3)	556 (3)	0.79 (0.01)	85 (1)
13	100	12_10	50	3	893 (7)	552 (2)	0.59 (0.03)	85 (1)
14	100	12_10	35	2	891 (11)	556 (5)	0.89 (0.01)	86 (1)
15	100	10_8	50	2	860 (6)	542 (5)	1.13 (0.03)	84 (1)
16	100	10_8	35	3	864 (22)	564 (6)	0.77 (0.06)	87 (2)
Average value for all variants					902 (21)	538 (16)	0.87 (0.19)	83 (2)

**Table 4:** Analysis of variance for selected factors and interactions between factors influencing DMax_FL_, ADMax_FL_, and DMin_CL_ of particleboards (SS—sum of squares; Df—degrees of freedom; MS—variance; F—Fisher’s F-test; *p*—significance level; X—factor influence).

Properties	Factor/Interaction	Statistical Measures					
		SS	Df	MS	F	*p*-Value	X
DMax_FL_	Content of the recovered particles (1)	10499	3	3500	46.07	0.000	52
	Resin load for face and core layer (2)	6460	1	6460	85.04	0.000	32
	Face layer ratio (3)	189	1	189	2.49	0.124	1
	Unit pressure (4)	18	1	18	0.24	0.626	0
	(1) × (2)	256	3	85	1.12	0.354	1
	(1) × (3)	195	3	65	0.86	0.473	1
	(1) × (4)	178	3	59	0.78	0.513	1
	Error	2431	32	76			12
DMin_CL_		SS	Df	MS	F	*p*-value	X
	Content of the recovered particles (1)	5242	3	1747	27.58	0.000	45
	Resin load for face and core layer (2)	14	1	14	0.22	0.641	0
	Face layer ratio (3)	2168	1	2168	34.22	0.000	19
	Unit pressure (4)	1722	1	1722	27.18	0.000	15
	(1) × (2)	235	3	78	1.23	0.313	2
	(1) × (3)	16	3	5	0.08	0.968	0
	(1) × (4)	154	3	51	0.81	0.498	1
	Error	2027	32	63			18
ADMax_FL_		SS	Df	MS	F	*p*-value	X
	Content of the recovered particles (1)	0.086	3	0.029	13.35	0.000	5
	Resin load for face and core layer (2)	0.466	1	0.466	217.21	0.000	26
	Face layer ratio (3)	0.043	1	0.043	19.85	0.000	2
	Unit pressure (4)	1.024	1	1.024	477.09	0.000	58
	(1) × (2)	0.020	3	0.007	3.06	0.042	1
	(1) × (3)	0.053	3	0.018	8.27	0.000	3
	(1) × (4)	0.010	3	0.003	1.51	0.231	1
	Error	0.069	32	0.002			4

## Data Availability

Data are contained within the article.
